# Long-term care units: a Portuguese study about the functional profile

**DOI:** 10.3389/fragi.2023.1192718

**Published:** 2023-05-04

**Authors:** César Fonseca, Ana Ramos, Bruno Morgado, Paulo Quaresma, José Garcia-Alonso, Anabela Coelho, Manuel Lopes

**Affiliations:** ^1^ São João de Deus Higher School of Nursing, University of Évora, Évora, Portugal; ^2^ Comprehensive Health Research Centre (CHRC), Universidade de Évora, Évora, Portugal; ^3^ Nursing Research, Innovation and Development Centre of Lisbon (CIDNUR), Nursing School of Lisbon (ESEL), Lisbon, Portugal; ^4^ Universidad Rovira i Virgili, Tarragona, Spain; ^5^ Department of Computer Science, University of Évora, Évora, Portugal; ^6^ Centro de Investigação e Desenvolvimento em Ciências Humanas e Sociais (CIDEHUS), University of Évora, Évora, Portugal; ^7^ Department of Computer and Telematics Systems, University of Extremadura, Badajoz, Spain; ^8^ Global Health and Tropical Medicine, Instituto de Higiene e Medicina Tropical, Universidade NOVA de Lisboa, Lisbon, Portugal

**Keywords:** aging, Portugal, long term care units, activities of daily living (ADL), functional status (MeSH), quality of health care (MeSH)

## Abstract

**Aim:** In this study, we analyze the relationship between the functional profile of older people admitted to long-term care units in Portugal and some demographic variables such as education level, sex, and age as well as the emotional state of mind.

**Methods:** A sample of 59,516 older people from the National Network of Integrated Continuous Care of Portugal were analyzed in this longitudinal study. All the retrospective data of the older people were collected during the period of hospitalization at the long-term care units. The database records of these units were analyzed, and a functional profile spanning the period of hospitalization was calculated.

**Results:** Activities of daily living and cognitive states improved, in the first 90 days of hospitalization, while mobility and instrumental activities of daily living worsened for the same period of 90 days. Generally, there was a decline in all domains after 450 days of hospitalization. The older women that did not attend school, those over 85 years old, and those who suffered from anxiety were pre-dominantly placed in the group of those with greater dependence (severe/complete dependence).

**Conclusion:** The participants hospitalized between 90 and 360 days presented the best results in the long-term care units of the National Network of Integrated Continuous Care of Portugal. With this study, we highlight the importance of evaluating the functional status of persons in long-term hospitalizations and the influence exerted by the level of education on the recovery and rehabilitation of dependence.

## 1 Introduction

Numerous changes occur in the biological domain with age, and these are the product of an accumulation of molecular and cellular damage, which translates into the progressive loss of the physiological reserves needed to face the aggressions of the environment and greater susceptibility to a decline in an individual’s intrinsic capacity (Urtamo et al., 2019).

In addition to all these physiological changes, there are also adjustments in the roles, social status, and ability to deal with the loss of close relationships. Motivation, life goals, and preferences tend to change as well, therefore aging can be a new state of learning for greater subjective wellbeing.

The aging of the population is a worldwide challenge considering the better response to the needs of the elderly, particularly in terms of healthcare. The statistical projections indicate a demographic profile, in terms of aging, that is unprecedented in history and is a product of the cumulative effect of the decrease in mortality and birth rates that has been occurring over the past several decades (OECD, 2019). According to Bao et al. (2019), multimorbidity, that is, suffering from more than one chronic disease, significantly increases the risk of dependence when combined with conditions that affect patients’ cognitive and mental status (Arco et al., 2021). Storeng et al. (2020) concluded in a cross-sectional analysis that people between the ages of 60 and 69 who suffer from three or more diseases fit into a complex morbidity profile that, over the years, reflects those who develop severe disabilities in carrying out their basic activities of daily living and have a moderate risk of mortality. The presence of multimorbidity or having visual, hearing, or mobility limitations cannot, by themselves, define the health condition of the older person. Disease classifications cannot capture all health statuses, as is the case of frailty in older people, with a prevalence of around 10%, which is characterized by a decrease in the individual homeostatic reserve to respond to endogenous and/or exogenous aggressions. This allows us to conclude that the functional profile/condition, as well as the health status of older people, cannot be established by the presence or absence of illness. All the circumstances of their life will interfere with their wellbeing, emotional state of mind, and other functional items. Studies more comprehensive will better demonstrate the impact of survival and quality of life predictors (Bao et al., 2019; Storeng et al., 2020; Arco et al., 2021).

Robine et al. (2019) argues that longevity is not synonymous with health, as well as living for many years does not necessarily graduate the quality of life. Societies more involved in the study of aging, chronic disease control, and the impact of the frailty of older adults have better results on healthy aging because they will improve social interactions, and their functional profiles and act as promoters of better cognitive status to achieve the “compression” of morbidity levels (Robine et al., 2019).

To integrate this need for continuous care and social support, the National Network of Integrated Continuous Care of Portugal was created in 2006 to support people who, regardless of age, fall into a situation of dependency. The Network intends to contribute to the quality of life, and to the consolidation of a fairer and more solidary society and involves the public and private sectors with the goal of rehabilitation, readaptation, and reintegration. It comprises different unit typologies, such as convalescence units, medium-term and rehabilitation units, long-term care units, integrated continuing care teams, inpatient units of pediatric integrated care, integrated continuing mental healthcare units, pediatric outpatient units, day units, and promotion of autonomy units (Pereira et al., 2021).

The long-term care unit aims to prevent and delay the worsening of patients’ de-pendency situations, providing maintenance care to advance comfort and quality of life. This unit is intended for people who need hospitalization for more than 90 consecutive days, and its typology ensures medical care, daily nursing care, drug administration, and prescription, supportive care such as physiotherapy, occupational therapy, psychosocial support, maintenance and stimulation activities, support in activities of daily living and in instrumental activities of daily living, care of hygiene, comfort, and food and promotion of conviviality and leisure. Care is provided 24 h a day, 7 days a week (Pereira et al., 2021). Based on the objectives of long-term care units, assessing their functionality is important to assessing health outcomes (Maresova et al., 2019; Ramos et al., 2022). The World Health Organization developed the International Classification of Functioning, Disability, and Health (ICF) to standardize the assessment of international functioning and disability related to the disease process, considering the structures and functions of the body and environmental factors (World Health Organization, 2001). The assessment of health status is an important indicator in determining care needs (World Health Organization, 2020; Coelho et al., 2022), and the literature suggests the assessment of several items, such as the self-perception of health status, limitations in basic life and instrumental activities, and mental health status (Hazra et al., 2018; Sicsic et al., 2020). Healthy aging encompasses a more global and holistic concept, in which all components of an older person’s life is valued. It is seen as a process of development and maintenance of functional capacity, which allows wellbeing in old age. The individual’s capacity does not remain constant over the years, it is shaped by life choices and interventions at different times, which determine the trajectory of each person. The World Health Organization (2020) declares 2021–2030 as the decade of healthy aging, to improve the quality of more years of life. The experience of healthy aging can be positive, depending on access to healthcare and the type of inclusion in a supportive environment (World Health Organization, 2020).

Self-care is a central concept in nursing care, defined as a regulatory human function, which refers to “the practice of activities that individuals initiate and perform for their own benefit, for the maintenance of life, health, and wellbeing” (Orem, 2001). When an individual cannot satisfy their self-care needs, they depend on someone or something for help and support, which means they are facing a self-care deficit (Orem, 2001; Martínez et al., 2021). The objective of the present study is to assess the functional profile of older people admitted to long-term care units in Portugal and to evaluate the relationship between the functional profile and other variables such as age, sex, education level, and emotional state of this population.

## 2 Materials and methods

The current study was a longitudinal retrospective study conducted in Portugal, including all the long-term units of the National Network of Integrated Continuous Care. Data were collected from 1 January 2008, to 27 February 2017.

### 2.1 Participants

The sample used in this study consisted of 59.516 older people aged 65 or over who were hospitalized in the long-term units of the National Network of Integrated Continuous Care in Portugal.

### 2.2 Instruments

The National Network of Integrated Continuous Care has its own information, registration, and monitoring system, which accommodates data from different sources such as hospitals, primary healthcare units, and social services. This system simultaneously shares information with different providers as an Integrated Assessment Instrument (IAI). The aim of this instrument (IAI) is to identify physical, functional, mental, and social disorders and life habits since its development in 2006, and all the results were crucial for the definition of an individual intervention plan that enhances the maintenance and recovery of capacities. The IAI as a multidimensional instrument allowed a diagnostic approach for early detection of care needs, with the aim of reducing morbidity and mortality.

The IAI includes the following constituent variables (Katz et al., 1963; Lawton, Brody; Folstein et al., 1975; Botelho, 1999).

• Demographic (sex, age, social status, and habits)
• Related to autonomy (physical and instrumental autonomy, locomotion, falls, and nutritional status)
• Related to complaints regarding health and emotional status
• Related to cognitive status as based on the Mini-Mental State.

The assessment of people’s health status was carried out by health professionals. All the variables support the conceptual map of self-care suggested by the literature (Ramos et al., 2017) and were validated in this study.

For easier analysis of results, age was divided into three age groups: 65–74 years old; 75–84 years, and 85 years or older.

The functional profile was analyzed through the following variables, extracted from the national records.

• Mobility and Walking such as walking on the street and going up and down stairs at home or inside buildings.
• Activities of Daily Living such as dressing, bathing, feeding, using the toilet, sphincter control, lying down and getting up, etc.
• Instrumental Activities of Daily Living such as taking medication, preparing meals, washing clothes, shopping, managing money, and using phones and transport.
• Cognitive State: cognitive processing of time and space.

To rate all these functional variables a Likert scale was used with the following description: no problem = 1; moderate problem = 2; severe problem = 3; and complete problem = 4.

To assure the validity and fidelity of the self-care variables and functional capacity items analysis of the main components were performed to Mobility (KMO = .743; *p* < .000); Activities of Daily Living (KMO = .885; *p* < .000); Instrumental Activities of Daily Living (KMO = .917; *p* < .000); Cognitive State (KMO = .593; *p* < .000) and Cronbach’s Alpha of α = 0.951 (Ramos et al., 2022), and all the outputs show excellent internal consistency. A high correlation was verified among the items constituting the Instrumental Activities of Daily Living. The items with the lowest (but still acceptable for the cognitive state) correlation were likely related to the lowest number of constituent items.

The impact of other variables on self-care, such as emotional state, was evaluated using the “emotional complaints” registered in IAI, which evaluates the amount of time they experienced feelings of anxiety and depressed mood: never = 1; shortly = 2; half of the time = 3; and longtime = 4.

The body mass Index was stratified as follows: <18.4 underweight; 18.5 to 25 suitable; >25, 1 overweight.

### 2.3 Data collection

Data were collected at the national level from the National Network of Integrated Continuous Care database, during the period between 1 January 2008, and 27 February 2017. These national records were compiled by health professionals, every 90 days, to assess the evolution of the functional profile of the patients that were in, the selected period, in a long-term care unit.

### 2.4 Statistical analysis

Principal Component Analysis allowed us to construct four indices: i) mobility, ii) basic life activities, iii) instrumental activities, and iv) cognitive state, through the statistical weighting of their indicators (using their factorial weights instead of the arithmetic mean), which gave them the designation of standardized values.

To analyze the four components of self-care capacity over the period of hospitalization, a longitudinal analysis was performed on the basis of parametric tests (one-way ANOVA and Student’s *t*-test).

We used the four components extracted in the Principal Component Analysis (mobility, basic life activities, instrumental activities, and cognitive state) and proceeded to their clustering into relatively homogeneous groups, through Cluster Analysis. First, the hierarchical method (Analyze Classify Hierarchical Cluster) was used to obtain information about the best solution, that is, the number of clusters to retain. Subsequently, the non-hierarchical optimization method was used, which compared to the first offers the advantage of greater accuracy in the classification, as well as being able to be applied in large databases, without restriction on the number of cases to be grouped simultaneously.

Considering that the data used was from big data, it was important to perform a random partition of the database to create a subsample with approximately 20% of the data and process the information by SPSS 25.

With Ward’s method, we obtained the agglomeration coefficients, and a graphic projection was performed of the highest of them (the final 30) to visualize their distances in order to identify that the best solution was the retention of 3 clusters. The cluster analysis was performed using the nonhierarchical optimization method available in IBM SPSS: K-means (Marôco, 2021).

### 2.5 Ethical procedures

The study was submitted and approved by the Ethics Committee of Scientific Research in the Areas of Human Health and Welfare of the University of Évora (report number, 17036; date of approval, 26 April 2017).

## 3 Results

### 3.1 Sociodemographic and clinical characteristics

The sample included 59,516 older people with an average age of 81.72 years. The majority were aged between 75 and 84 years (44.5%), were female (57.8%), were married (45.7), and had completed between 1 and 6 years of schooling (51.9%) (Table 1).

**TABLE 1:** Sociodemographic data from the long-term care unit.

Sociodemographic variables	n (%)	Sociodemographic variables	n (%)
**Age (years)**	10591 (17.8)	**Education (years)**	14419 (42.7)
65–74	26498 (44.5)	None	17513 (51.9)
75–84	22427 (37.7)	1 to 6	962 (2.8)
≥85		7 to 12	869 (2.6)
		≥13	
**Sex**	34400 (57.8)	**Professional level**	24687 (73.2)
Female	25116 (42.0)	Professional	7304 (21.6)
Male		Nonprofessional	1348 (4.0)
		Intermediate	406 (1.2)
		Specialist	
**Marital Status**	7420 (13.8)	**Region of Residence**	5584 (10.1)
Single	24609 (45.7)	Alentejo	2517 (4.5)
Married	167 (0.3)	Algarve	15632 (28.1)
Unmarried Partner	1598 (3.0)	Centro	15847 (28.5)
Divorced	19988 (37.1)	Lisboa e Vale do Tejo	15982 (28.8)
Widower	97 (0.2)	Norte	
Unknown			

### 3.2 Functional profile evolution

**FIGURE 1:**
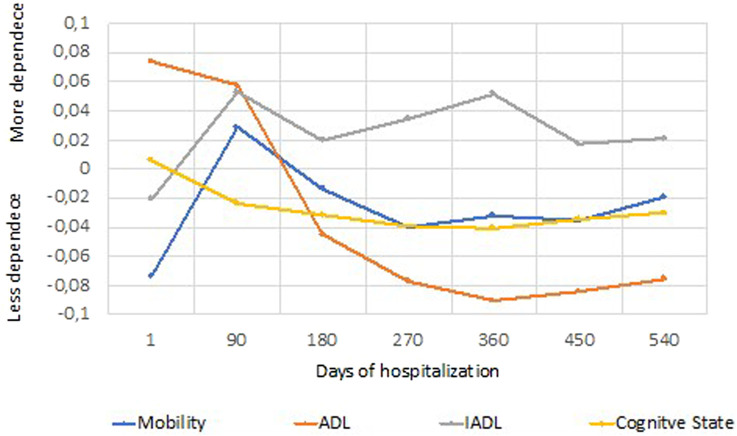
Synthetic indices: Mobility, ADL (activities of daily living), IADL (instrumental activities of daily living) and cognitive status in the long-term care unit (means of standardized values).

The population’s functional profile evolution throughout their hospitalization is shown in Figure 1. The *x*-axis refers to the days of hospitalization of older people (it is mandatory to have a systematic assessment of the health condition every 90 days following admission). The *y*-axis presents the average of the standardized values, in which the scores should be interpreted as high values (>0.04) = higher degree of dependence; moderate values (−0.02 to 0.02) = moderate degree of dependence; low values (<-0.04) = lower degree of dependence.

Statistically significant differences were observed in the cognitive status [F (119.324163) = 10.369; *p* < 0.001], the mobility dependence profile [F (119.324168) = 12.636; *p* < 0.001], and the activities of daily living such as the basic [F (119.328779) = 19.937; *p* < 0.001] as well as the instrumental [F (95.71842) = 7.767; *p* < 0.001].

Throughout the hospitalization period, the highest mortality rate was recorded between the first evaluation (admission) and the second evaluation: 10.4% (*N* = 12180) in Long Duration and Maintenance Units. This finding shows that the phase of greatest exacerbation or decompensation of the chronic disease occurs in the initial phase of hospitalization.

### 3.3 Dependence clusters

**FIGURE 2:**
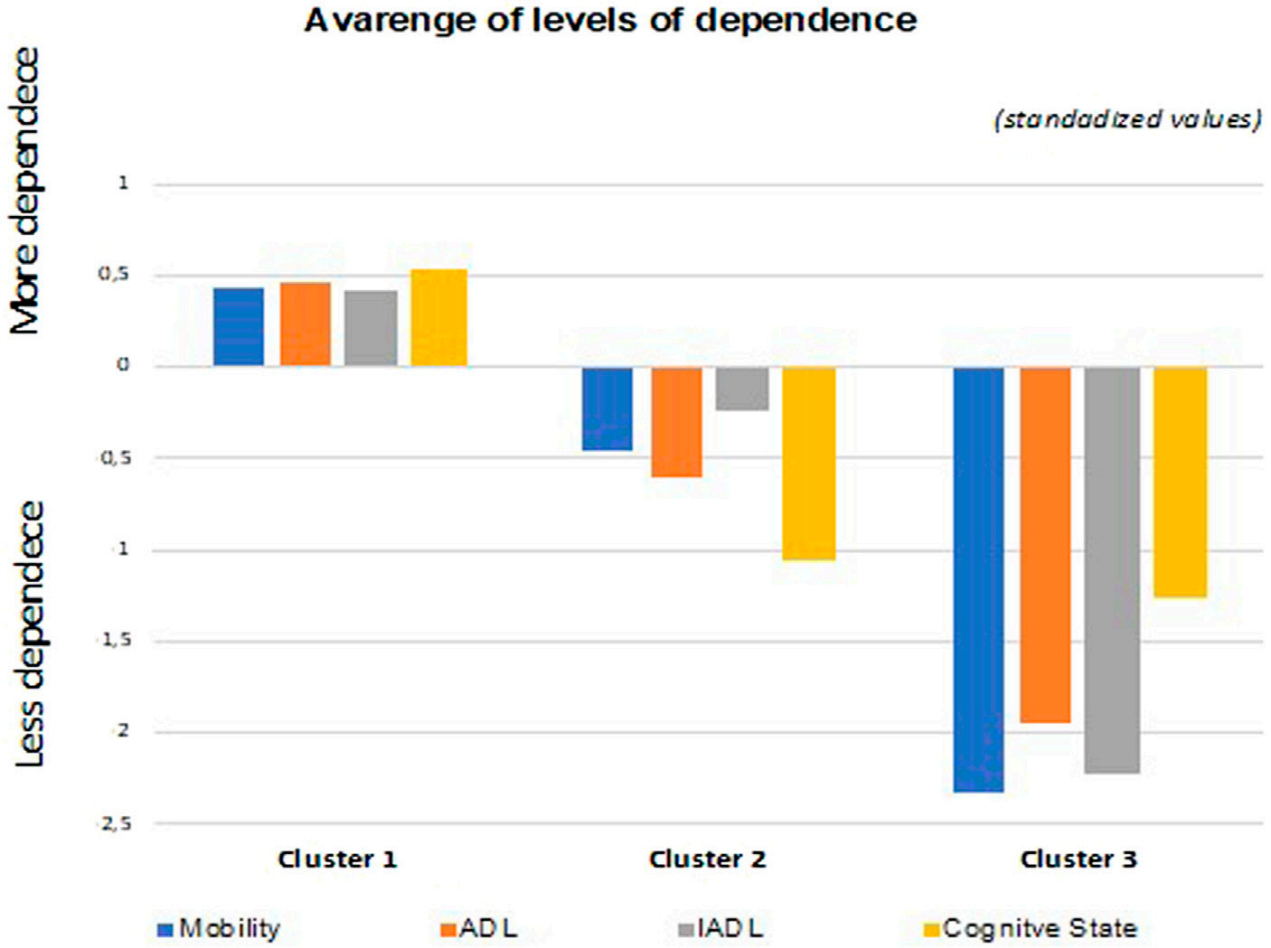
Average dependency levels of Cluster 1, Cluster 2, and Cluster 3 regarding the variables of mobility, activities of daily living (ADL), instrumental activities of daily living (IADL), and cognitive status in the long-term care unit.

Figure 2 shows the grouping of the participants as follows: Cluster 1%–61.1% (*N* = 41188); Cluster 2%–30.8% (*N* = 20777); and Cluster 3%–8.1% (*N* = 5,440). Statistically significant differences were detected in the items related to mobility [F (2.67402) = 46106.824; *p* < 0.001], basic life activities [F (2.67402) = 45130.370; *p* < 0.001], instrumental activities [F (2.67402) = 36870.863; *p* < 0.001] and cognitive status [F (2.67402) = 50425.168; *p* < 0.001]. The assessment was carried out during the admission.

The three clusters, in average terms, comprise the following.

• Cluster 1: Older people with a higher degree of dependence (severe or complete self-care deficit);
• Cluster 2: Older people with an intermediate degree of dependence (moderate self-care deficit);
• Cluster 3: Older people a lower degree of dependence (low self-care deficit).

**FIGURE 3:**
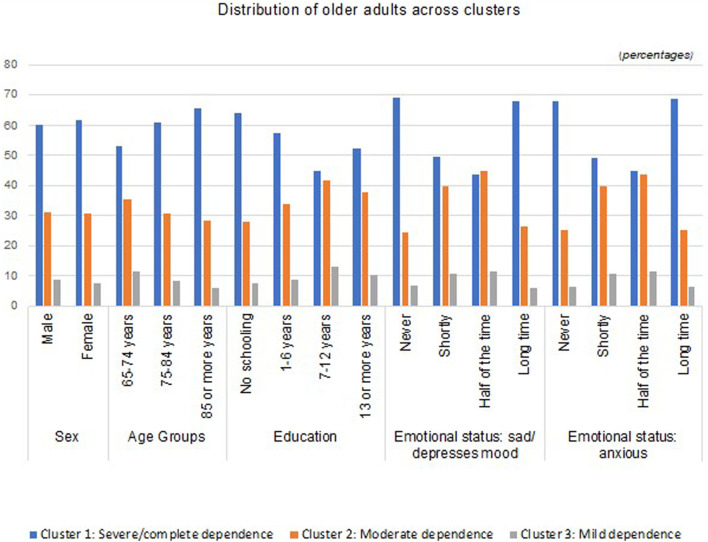
Distribution of clusters (people aged ≥65 years old hospitalized in the long-term care unit) regarding the sex, age group, educational level, levels of depression, and levels of anxiety per cluster.

Figures 3 show the differences between the three clusters and the variables of sex, age, education, depression, and anxiety.

• Cluster 1 (severe/complete dependence) comprises a higher percentage of females aged 85 or over. It is formed by people who did not go to school, who are underweight, who experience long periods of anxiety, and who do not experience depression;
• Cluster 2 (moderate dependence) includes a greater number of females, aged between 65 and 84 years, with 7–12 years of schooling, with a normal body mass index, and who experience depression or anxiety about half of the time;
• Cluster 3 (low dependence) is primarily composed of males aged between 65 and 74 years, with 7 or more years of schooling, with a normal body mass index, and who experience depression or anxiety about half of the time.

## 4 Discussion

This study evaluated the functional profile and evolution of older people hospitalized in the long-term care units of the National Network of Integrated Continuous Care in Portugal and then related that profile to sex, level of education, and emotional state. In the first 90 days of hospitalization following admission, we observed an increase in both mobility dependency and performance among instrumental activities, as well as an improvement in the cognitive status and basic activities of daily living. However, recovery is evident after 180 days (2nd follow-up) in all domains. It should be noted that from 360 to 450 days, the dependence on activities of daily living and the cognitive state both begin to deteriorate. The degree of dependence on the basic activities of daily living is not exclusively determined by mobility because of the strong influence exerted by the cognitive state in this process, so these results are not surprising (de Oliveira Silva et al., 2019; Lovett et al., 2020). After 450 days, all domains declined, increasing the degree of dependence. It must be considered that this type of care is intended for people with chronic diseases that have a high degree of complexity, for whom this care cannot be provided at home (Administração Central do Sistema de Saúde ACSS, 2014; Administração Central do Sistema de Saúde ACSS, 2016). These data are in line with studies that demonstrate that during hospitalization, older people with compromised mobility tend to worsen at the beginning of their stay in terms of carrying out their activities of daily living, but during the period of hospitalization in long-term care units, these patients tend to show an improvement with physical rehabilitation (Crocker et al., 2013; Cuevas-Lara et al., 2021). However, an analysis of the study carried out in convalescence units shows an improvement in all domains in the first 30 days (Ramos et al., 2021). Therefore, we can infer the importance of correctly assessing people’s needs and rehabilitation potential prior to referral to an integrated continued care unit. If people have the potential for a quick recovery, then they should be referred to convalescence units; on the other hand, if more time is needed for rehabilitation or they just need the care to sustain their wellbeing, then they should be referred to a long-term care unit.

When it is not possible to revert the situation of severe/complete dependency and people do not have family support or resources in their homes to deal with their care needs we have other options, outside the Network, such as the residential structures. In Portugal, residential structures are not included in the LTC, but they can guarantee the continuity of care under an institutionalization regime (Pereira et al., 2021).

In terms of age, individuals who are over 85 years old are in the severe/complete dependence group, the group between 65 and 84 years old are in the moderate dependence group and most individuals between 65 and 74 years of age are in the mild dependence group, which is in line with studies that show that older people have poor functionality (Yeh et al., 2014; Micheli et al., 2018). The same fact was verified when analyzing global data from the National Network of Integrated Continuous Care (Convalescence Units) in Portugal (Ramos et al., 2021).

When analyzing the clusters by sex, there is a higher percentage of females in the “Severe/Complete” and “Moderate” clusters, while males are more prevalent in the mild dependence cluster. These findings should be carefully analyzed due to the contradictory findings that can be found in the literature. We found studies that confirm these results, assuming there are no great differences between the sexes in ages above 65 years and concluding that sex is not a major predictor of dependence levels (James et al., 2018; Ek et al., 2021). On the other hand, we have also found evidence from other authors concluding that females experience higher levels of functional dependence (Schmitz and Lazarevič, 2020; Fonseca et al., 2021; Lopes et al., 2021). It is necessary to take into account the fact that females live longer, so reaching more advanced stages of life gives them a degree of dependence that men do not tend to reach because they die earlier, which is a phenomenon referred to as the “feminization of old age” (Goes et al., 2020).

Regarding the level of education, the results indicate that people without formal education more predominately fall into the “Serious/Complete” dependency cluster, while most older people with 7 years of school education or more fall into the “Moderate” and “Slight” dependency clusters. There are several recent studies that corroborate these results, indicating that lower levels of education are associated with higher levels of dependence (Serrano-Alarcón and Perelman, 2017; Abalo et al., 2018; Coutinho et al., 2018; Cui et al., 2019). In addition, a Portuguese study that evaluated the functional and cognitive profiles of both the institutionalized and noninstitutionalized older population, as well as the consequence of sociodemographic factors on functionality and cognition, concluded that the lack of education is one of the main predictors of decreased functionality at elderly ages (Lopes et al., 2021).

An analysis of emotional states suggests that older people who experience long periods of anxiety predominately fall in the “severe/complete” dependency cluster, and individuals who experience lower rates of depression and anxiety tended to fall into the moderate and mild dependency cluster. A study that sought to investigate the relationship between multimorbidity and dependence in older people concluded that the risk of dependence is higher for participants who suffer from depression than for those who do not (Quiñones, Markwardt, Thielke, Rostant, Vásquez, Botoseneanu; Morgado et al., 2021). Although we have not concretely evaluated the diagnosis of depression, we have evaluated symptoms, such as anxiety and sadness, that are part of the depressive symptomatology. In fact, studies indicate that the mental health conditions associated with other chronic conditions serve to increase dependence in hospitalized older people (James et al., 2018; McClellan et al., 2021), which influences functionality (Pinho et al., 2017; Pinho et al., 2020). Activities of Daily Living and Instrumental Activities of Daily Living disabilities were associated with depressive symptoms even after controlling people’s sociodemographic characteristics, welfare, health conditions, and informal care (Zhao et al., 2022).

According to Doroszkiewicz (2022) the socio-demographic variables of older people (such as sex, age, marital status, dwelling typologies, self-evaluation of health, and loneliness), their physical functional status (according to the Barthel scale) and the Instrumental Activities of Daily Living (such as locomotion, vision and hearing, emotional and cognitive state, and risk of falls or pressure sores) are related to the self-care dependency. The results of this study show that the increasing impairment of cognitive functions in older people has an important impact on the care-dependency level (Doroszkiewicz, 2022). The most prevalent healthcare needs in people with moderate or severe dementia and moderate or severe frailty were food preparation, medication/taking pills, looking after their home, toilet use, sensory disabilities, memory, communication and social interaction, bowels, sleeping, feeding and drinking and falls prevention (Abreu et al., 2019).

Being overweight or obese may cause many chronic illnesses. Furthermore, several studies have shown that high body mass index is associated with mortality and morbidity among older people. However, some studies reported the opposite and conclude that obesity is protective against Activities of Daily Living. The study done by Ozturk et al. (2017) showed increased values of body mass index and Activities of Daily Living ability, as this study, which could be indicative of protective effects.

On the other hand, being underweight was associated with an impaired Mini-mental State score and worse performance in Instrumental Activities of Daily Living. Also, being overweight was associated with a better score on the Mini-mental State and the Instrumental Activities of Daily Living. For both outcomes education level significantly influenced the predictions (Borda et al., 2021).

From the global analysis of the data obtained, the recovery of the ability to walk and the improvement in performance in basic activities of daily living were clearly the most evident achievements, compared to instrumental activities and cognitive state. These findings emphasize the importance of memory and complex reasoning for carrying out instrumental activities, such as going shopping, managing money, doing housework, or using the telephone, among others. Thus, when there is a deficit in carrying out instrumental activities, there is a high possibility of a decline in mental and cognitive functions, and their restitution is difficult and slow, which explains their shorter recovery curve. The study carried out shows the close relationship between the determinants of self-care ability, such as older age, low education, low body mass index, and depressed and/or anxious mood, which contribute to a greater degree of dependence, as reported in those studies that are explained. Dependence on self-care can be permanent or temporary, which means that it can be prevented, reduced, or reversed since that exists an appropriate environment and assistance.

## 5 Conclusion

In the first 90 days of hospitalization in a long-term care unit, an increase in dependence was verified relating to both mobility and Instrumental Activities of Daily Living, while a decrease in dependence relating to both cognitive state and Activities of Daily Living was also seen. Hospitalization in the long-term care unit was shown to result in functionality gains; however, after 450 days of hospitalization, dependence worsened across all domains. We also conclude that in the severe or complete dependence cluster, women, older people over the age of 85, and those with no education are more prevalent. This study thus highlights the importance of schooling and literacy for recovery and rehabilitation from an acute illness or a chronic disease exacerbation and for the regaining of independence. Strategies should therefore be delineated to promote the population’s focus on active and healthy aging and the best ways to recover from various levels of dependence.

With this study, we also point out the importance of developing future studies to evaluate the effectiveness of interventions that promote the mental health of older people as a structural axis for improving their functional capacity and level of self-care. Further research is also recommended to study the weight of other determinants (pressure ulcers; polypharmacy; pain; hydration status; respiratory status; consumption of psychotropic drugs; physical restriction/mechanical restraint; health literacy and infections) on functional capacity as well as the study of the potential of analytical information extracted from electronic health systems and records in the quality of long-term care.

## 6 Limitations, and strengths

This study represents a relevant contribution to the older population and health services because by knowing the predictors of dependency in self-care it is possible to outline health policies and early, individualized, and appropriate interventions for the preservation of their functional capacity.

The study carried out has several limitations related to the study design once researchers could not control exposure or outcomes and must rely on records that others have made. The retrospective collection of data up to 2017 from a convenience sample will not represent the general population and would benefit from recent data in order to analyze other variables such as the COVID-19 pandemic impact.

Another limitation of the present study is the lack of assessment of the frailty condition of older people and it should be considered in further research once has a high impact on the dependence profile in activities of daily living.

## Data Availability

The raw data supporting the conclusion of this article will be made available by the authors, without undue reservation.

## References

[B1] Abalo E. M. Mensah C. M. Agyemang-Duah W. Peprah P. Budu H. I. Gyasi R. M. (2018). Geographical differences in perceived health status among older adults in Ghana: Do gender and educational status matter? Gerontology Geriatric Med. 4, 2333721418796663. 10.1177/2333721418796663 PMC6124182 30202775

[B2] Abreu W. Tolson D. Jackson G. A. Staines H. Costa N. (2019). The relationship between frailty, functional dependence, and healthcare needs among community-dwelling people with moderate to severe dementia. Health and Soc. care community 27, 642–653. 10.1111/hsc.12678 30402986

[B3] Administração Central do Sistema de Saúde (Acss) (2014). Monitorização da rede nacional de cuidados continuados integrados (rncci) . http://www.acss.min-saude.pt/Portals/0/Relat%C3%B3rio%20de%20Monitoriza%C3%A7%C3%A3o%20Anual%20da%20RNCCI%202014.pdf (Accessed March 15, 2023).

[B4] Administração Central do Sistema de Saúde (Acss) Monitorização mensal da RNCCI: Janeiro de 2016. Available online: https://www.sns.gov.pt/monitorizacao-do-sns/gestao-recursos-sns/gestao-recursos-cci/ (Accessed March 15, 2023).

[B5] Arco H. Pedro A. Pinho L. Proença A. (2021). “Aging and functionality of the institutionalized elderly people of Alto Alentejo: Contributions to the diagnosis of the situation,” in Gerontechnology III: Contributions to the Third International Workshop on Gerontechnology, IWoG 2020, Évora, Portugal, October 5–6, 2020. Editor Fonseca C. García-Alonso J. (Springer, Cham: Springer Nature. Lecture Notes in Bioengineering), 87–98.

[B6] Bao J. Chua K.-C. Prina M. Prince M. (2019). Multimorbidity and care dependence in older adults: A longitudinal analysis of findings from the 10/66 study. BMC Public Health 19, 585. 10.1186/s12889-019-6961-4 31096943 PMC6524243

[B7] Borda M. G. Venegas-Sanabria L. C. Garcia-Cifuentes E. Gomez R. C. Cano-Gutierrez C. A. Tovar-Rios D. A. (2021). Body mass index, performance on activities of daily living and cognition: Analysis in two different populations. BMC Geriatr. 21, 177. 10.1186/s12877-021-02127-8 33711937 PMC7953600

[B8] Botelho M. (1999). Autonomia Funcional em Idosos: Caracterização multidimensional em idosos utentes de um centro de saúde urbano. Universidade Nova de Lisboa.Lisbon, Portugal.

[B9] Coelho A. de Bienassis K. Klazinga N. Santo S. Frade P. Costa A. (2022). Mental health patient-reported outcomes and experiences assessment in Portugal. Int. J. Environ. Res. Public Health 19 (18), 11153. 10.3390/ijerph191811153 36141427 PMC9517602

[B10] Coutinho A. T. D. Q. Vilela M. B. R. Lima M. L. L. T. (2018). Social communication and functional independence of the elderly in a community assisted by the family health strategy. Rev. CEFAC 20, 363–373. 10.1590/1982-0216201820313417

[B11] Crocker T. Young J. Forster A. Brown L. Ozer S. Greenwood D. C. (2013). The effect of physical rehabilitation on activities of daily living in older residents of long-term care facilities: Systematic review with meta-analysis. Age Ageing 42, 682–688. 10.1093/ageing/aft133 24004604

[B12] Cuevas-Lara C. Izquierdo M. Sáez de Asteasu M. L. Ramírez-Vélez R. Zambom-Ferraresi F. Zambom-Ferraresi F. (2021). Impact of game-based interventions on health-related outcomes in hospitalized older patients: A systematic review. J. Am. Med. Dir. Assoc. 22, 364–371.e1. 10.1016/j.jamda.2020.07.027 32873472

[B13] Cui S. Wang R. Lu L. Wang H. Zhang Y. (2019). Influence of education level on mental health and medical coping modes: A correlation analysis in the elderlies. Am. J. Nurs. Sci. 8, 324. 10.11648/j.ajns.20190806.16

[B14] de Oliveira Silva F. Ferreira J. V. Plácido J. Sant’Anna P. Araújo J. Marinho V. (2019). Three months of multimodal training contributes to mobility and executive function in elderly individuals with mild cognitive impairment, but not in those with alzheimer’s disease: A randomized controlled trial. Maturitas 126, 28–33. 10.1016/j.maturitas.2019.04.217 31239114

[B15] Doroszkiewicz H. (2022). How the cognitive status of older people affects their care dependency level and needs: A cross-sectional study. Int. J. Environ. Res. public health 19, 10257. 10.3390/ijerph191610257 36011890 PMC9408506

[B16] Ek S. Rizzuto D. Xu W. Calderón-Larrañaga A. Welmer A.-K. (2021). Predictors for functional decline after an injurious fall: A population-based cohort study. Aging Clin. Exp. Res. 33, 2183–2190. 10.1007/s40520-020-01747-1 33161531 PMC8302494

[B17] Folstein M. F. Folstein S. E. McHugh P. R. (1975). Mini-mental state. A practical method for grading the cognitive state of patients for the clinician. J. psychiatric Res. 12, 189–198. 10.1016/0022-3956(75)90026-6 1202204

[B18] Fonseca C. de Pinho L. G. Lopes M. J. Marques M. do C. Garcia-Alonso J. (2021). The elderly nursing core set and the cognition of Portuguese older adults: A cross-sectional study. BMC Nurs. 20, 108. 10.1186/s12912-021-00623-1 34162387 PMC8220736

[B19] Goes M. Lopes M. J. Oliveira H. Fonseca C. Marôco J. (2020). A nursing care intervention model for elderly people to ascertain general profiles of functionality and self care needs. Sci. Rep. 10, 1770. 10.1038/s41598-020-58596-1 32019984 PMC7000781

[B20] Hazra N. C. Rudisill C. Gulliford M. C. (2018). Determinants of health care costs in the senior elderly: Age, comorbidity, impairment, or proximity to death? Eur. J. Health Econ. 19, 831–842. 10.1007/s10198-017-0926-2 28856487 PMC6008359

[B21] James S. L. Abate D. Abate K. H. Abay S. M. Abbafati C. Abbasi N. (2018). Global, regional, and national incidence, prevalence, and years lived with disability for 354 diseases and injuries for 195 countries and territories, 1990–2017: A systematic analysis for the global burden of disease study 2017. Lancet 392, 1789–1858. 10.1016/s0140-6736(18)32279-7 30496104 PMC6227754

[B22] Katz S. Ford S. Moskowitz R. Jackson B. Jaffe M. (1963). Studies of illness in the aged: The index of ADL, a standardized measure of biological and psychosocial function. JAMA,9 185. 10.1001/jama.1963.03060120024016.185:12 14044222

[B23] Lawton M. Brody E. (1969). Assessment of older people: Self‐maintaining and instrumental activities of daily living. Gerontologist 9 (3), 179–186. 10.1093/geront/9.3_part_1.179 5349366

[B24] Lopes M. J. Pinho L. G. D. Fonseca C. Goes M. Oliveira H. Garcia-Alonso J. (2021). Functioning and cognition of Portuguese older adults attending in residential homes and day centers: A comparative study. Int. J. Environ. Res. Public Health 18, 7030. 10.3390/ijerph18137030 34209339 PMC8297339

[B25] Lovett R. M. Curtis L. M. Persell S. D. Griffith J. W. Cobia D. Federman A. (2020). Cognitive impairment no dementia and associations with health literacy, self-management skills, and functional health status. Patient Educ. Couns. 103, 1805–1811. 10.1016/j.pec.2020.03.013 32197929 PMC7864102

[B26] Maresova P. Javanmardi E. Barakovic S. Barakovic Husic J. Tomsone S. Krejcar O. (2019). Consequences of chronic diseases and other limitations associated with old age – A scoping review. BMC Public Health 19, 1431. 10.1186/s12889-019-7762-5 31675997 PMC6823935

[B27] Marôco J. (2021). Análise Estatística com o SPSS Statistics v18–v27. 8th. Lisboa, Portugal: Pêro Pinheiro.

[B28] Martínez N. Connelly C. D. Pérez A. Calero P. (2021). Self-care: A concept analysis. Int. J. Nurs. Sci. 8 (4), 418–425. 10.1016/j.ijnss.2021.08.007 34631992 PMC8488814

[B29] McClellan S. P. Haque K. García-Peña C. (2021). Diabetes multimorbidity combinations and disability in the Mexican health and aging study. Archives Gerontology Geriatrics 93, 104292. 10.1016/j.archger.2020.104292 PMC7887040 33186887

[B30] Micheli K. Ratsika N. Vozikaki M. Chlouverakis G. Philalithis A. (2018). Family ties and functional limitation in the elderly: Results from the survey of health ageing and retirement in europe (SHARE). Archives Gerontology Geriatrics 78, 23–29. 10.1016/j.archger.2018.05.023 29883806

[B31] Morgado B. Fonseca C. Lopes M. Pinho L. (2021). Components of care models that influence functionality in people over 65 in the context of long-term care: Integrative literature review. Lect. Notes Bioeng., 324–335. 10.1007/978-3-030-72567-9_30

[B32] Oecd (2019). Health at a glance 2019: OECD indicators. Paris, France: OECD Publishing.

[B33] Orem D. N. (2001). Concepts of practice. 6th. St Louis, MO, USA: Mosby.

[B34] Ozturk G. Z. Egici M. T. Bukhari M. H. Toprak D. (2017). Association between body mass index and activities of daily living in homecare patients. Pak. J. Med. Sci. 33, 1479–1484. 10.12669/pjms.336.13748 29492082 PMC5768848

[B35] Pereira C. Fonseca C. Pinho L. (2021). “A rede nacional de Cuidados continuados integrados em Portugal,” in Os Cuidados de Saúde Face Aos Desafios do Nosso Tempo: Contributos para a Gestão da Mudança. Évora. Editors Lopes M. Sakellaridesm C. (Lisboa, Portugal: Universidade de Évora), 36–47.

[B36] Pinho L. G. de Pereira A. Chaves C. (2017). Influence of sociodemographic and clinical characteristics on the quality of life of patients with schizophrenia. Rev. Esc. Enferm. USP 51, e03244. 10.1590/s1980-220x2016031903244 28902324

[B37] Pinho L. G. Pereira A. Chaves C. Sequeira C. Sampaio F. Correia T. (2020). Affectivity in schizophrenia: Its relations with functioning, quality of life, and social support satisfaction. J. Clin. Psychol. 76, 1408–1417. 10.1002/jclp.22943 32072643

[B38] Quiñones A. R. Markwardt S. Thielke S. Rostant O. Vásquez E. Botoseneanu A. (2017). Prospective disability in different combinations of somatic and mental multimorbidity. Journals Gerontology Ser. A 73, 204–210. 10.1093/gerona/glx100 PMC6279134 28541396

[B39] Ramos A. Fonseca C. Henriques A. (2017). Needs of fundamental care in elderly with dependence on self-care in context of long-term care: A scoping review. Int. J. Curr. Res. 9, 7.

[B40] Ramos A. Fonseca C. Pinho L. Lopes M. Brites R. Henriques A. (2022). Assessment of functioning in older adults hospitalized in long-term care in Portugal: Analysis of a big data. Front. Med. 9, 780364. 10.3389/fmed.2022.780364 PMC8964623 35372382

[B41] Ramos A. Fonseca C. Pinho L. Lopes M. Oliveira H. Henriques A. (2021). Functional profile of older adults hospitalized in convalescence units of the national Network of integrated continuous care of Portugal: A longitudinal study. J. Personalized Med. 11, 1350. 10.3390/jpm11121350 PMC8704872 34945822

[B42] Robine J. M. (2019). “Successful aging and the longevity revolution,” in The cambridge handbook of successful aging. Editors Fernandez-Ballesteros R. Benetos A. Robine J. M. (Cambridge, London: Cambridge University Press), 27–38.

[B43] Schmitz A. Lazarevič P. (2020). The gender health gap in europe’s ageing societies: Universal findings across countries and age groups? Eur. J. Ageing 17, 509–520. 10.1007/s10433-020-00559-6 33376463 PMC7752938

[B44] Serrano-Alarcón M. Perelman J. (2017). Ageing under unequal circumstances: A cross-sectional analysis of the gender and so-cioeconomic patterning of functional limitations among the southern European elderly. Int. J. Equity Health 16, 175. 10.1186/s12939-017-0673-0 28974223 PMC5627490

[B45] Sicsic J. Ravesteijn B. Rapp T. (2020). Are frail elderly people in europe high-need subjects? First evidence from the SPRINTT data. Health Policy 124, 865–872. 10.1016/j.healthpol.2020.05.009 32507482

[B46] Storeng S. H. Vinjerui K. H. Sund E. R. Krokstad S. (2020). Associations between complex multimorbidity, activities of daily living and mortality among older Norwegians. A prospective cohort study: The hunt study, Norway. BMC Geriatr. 20, 21. 10.1186/s12877-020-1425-3 31964341 PMC6974981

[B47] Urtamo A. Jyväkorpi S. K. Strandberg T. E. (2019). Definitions of successful ageing: A brief review of a multidimensional concept. Acta bio-medica 90, 359–363. 10.23750/abm.v90i2.8376 31125022 PMC6776218

[B48] World Health Organization (2020). Decade of healthy ageing: Baseline report. Geneva, Switzerland: World Health Organization. https://apps.who.int/iris/handle/10665/338677.

[B49] World Health Organization (2001). The international classification of functioning, disability and health (ICF). Geneva, Switzerland: World Health Organization.

[B50] Yeh K. Lin M. Liu L. Chen L. Peng L. Chen L. (2014). Functional decline and mortality in long-term care settings: Static and dynamic approach. J. Clin. Gerontology Geriatrics 5, 13–17. 10.1016/j.jcgg.2013.08.001

[B51] Zhao L. Wang J. Deng H. Chen J. Ding D. (2022). Depressive symptoms and ADL/IADL disabilities among older adults from low-income families in dalian, liaoning. Clin. Interv. Aging 17, 733–743. 10.2147/CIA.S354654 35574289 PMC9091470

